# The Dread of Death

**Published:** 1889-11

**Authors:** 


### THE DREAD OF DEATH.

Sir Lyon Playfair, in a letter to Junius Henri Browne, author of a paper with the above title, says: "Having represented a large medical constituency (the University of Edinburgh) for seventeen years as a member of Parliament, I naturally came in contact with the most eminent medical men in England. I have put the question to most of them, 'Did you, in your extensive practice, ever know a patient who was afraid to die?' With two exceptions they answered 'No.' One of these exceptions was Sir Benjamin Brodie, who said he had seen one case. The other was Sir Robert Christian, who had seen one case, that of a girl of bad character, who had a sudden accident. I have known three friends who were partially devoured by wild beasts under apparently hopeless circumstances of escape. The first was Livingstone, the great African traveler, who was knocked on his back by a lion, which began to munch his arm. He assured me that he felt no fear or pain, and that his only feeling was one of intense curiosity as to which part of his body the lion would take next. The next was Rustem Pasha, now Turkish Ambassador in London. A

bear attacked him, and tore off part of his arm and shoulder. He also assured me that he had neither pain nor fear, but that he felt excessively angry because the bear grunted with so much satisfaction in munching him. The third case is that of Sir Edward Bradford, an Indian officer, now occupying a high position in the Indian office. He was seized in a solitary place by a tiger, which held him firmly behind the shoulders with one paw, and then deliberately devoured the whole of his arm, beginning at the end and ending at the shoulder. He was positive that he had no sensation of fear, and thinks that he felt a little pain when the fangs went through his hand, but is certain that he felt none during the munching of his arm."—*The New Idea.*

